# Linkage Between Poverty and Smoking in Philadelphia and Its Impact on Future Directions for Tobacco Control in the City

**DOI:** 10.1089/pop.2019.0006

**Published:** 2020-01-30

**Authors:** Christine S. Shusted, Gregory C. Kane

**Affiliations:** ^1^ Department of Medicine, Thomas Jefferson University, Philadelphia, Pennsylvania.; ^2^ Division of Pulmonary and Critical Care, Department of Medicine, The Jane and Leonard Korman Respiratory Institute, Sidney Kimmel Medical College at Thomas Jefferson University, Philadelphia, Pennsylvania.

**Keywords:** smoking, poverty, social ecological model, multifaceted factors, tobacco control

## Abstract

Poverty is linked to negative health consequences and harmful health behaviors such as smoking. Despite this established correlation, few comparative studies have investigated the relationship between local poverty rates and smoking in urban settings through a Social Ecological Model framework. The authors sought to examine the linkage between local poverty rates in Philadelphia, Pennsylvania and adult smoking rates by scrutinizing existing patterns and potential mediating factors via publicly accessible data in established planning districts. The authors determined several individual, interpersonal, organizational, community, and environmental factors, varying across these districts, that impact smoking in Philadelphia. Poverty rates influence the resources, demographic makeup, and number of tobacco retailers a district has, which have downstream effects. The authors recommend that further investment is allocated to planning districts in order to mitigate the risk of smoking.

## Introduction

Philadelphia, Pennsylvania is currently the poorest large city in the United States with a poverty rate of 25.8%, which equates to 400,000 impoverished residents.^1–3^ Poverty in Philadelphia is widespread and pervasive, and the city has some of the worst income disparities in the country. The city has the third worst income gap in the country.^4^ Philadelphia's poverty rates vary drastically by neighborhood. One quarter of the census tracts have a poverty rate of at least 38%, while another quarter have a poverty rate of less than 13%.^5^

Poverty can predispose individuals to several adverse health outcomes such as premature mortality as noted by the health–wealth gradient. There is a significant relationship between poverty and risky health behaviors such as a sedentary lifestyle, malnutrition, and tobacco use.^6^ Philadelphia's high poverty rate places its residents at risk for negative health outcomes and unhealthy behaviors.

Smoking can lead to several adverse health outcomes including lung and esophageal cancer, as well as several other diseases that contribute to premature death.^7^ Philadelphia has the highest adult smoking rate of the large cities in the country.^1^ In 2015, the adult smoking rate was 22.4%, higher than the national rate of 16.8%.^1^ Although higher than other cities, the prevalence of adult smoking in Philadelphia has declined over recent years. In 2008, the smoking rate peaked at 27.3% and steadily has waned since, flatlining at 22.4% around 2014.^2^ Philadelphia had a population of 1.58 million in 2017; meaning if the smoking rate continues to hold, roughly 354,000 adult residents in the city smoke.^1–3,8^

In addition to high smoking rates, Philadelphia has the highest incidence of lung cancer in the state; at a rate of 78.7 per 100,000 cases, well over the national average of 60.2 and the state average of 64.7.^9^ Tobacco use is the leading cause of death in Philadelphia, with the smoking-attributable mortality rate averaging 468 per 100,000 deaths.^2^ Smoking also costs the city economically; annually, the city loses $675 million in productivity related to smoking-attributable diseases.^10^ Philadelphia attributes the high rates of smoking, smoking-related disease burden, mortality, and loss of productivity to high levels of poverty, inexpensive cigarettes, smoking norms, and the availability of cigarettes.^10^

The research team set out to examine how poverty and smoking are related within the city utilizing publicly available data from the Philadelphia Department of Public Health, the 2012–2015 Public Health Management Corporation Household Health Survey, the Pennsylvania Department of Education, and the US Census Bureau.^1,2,11–13^ The team also explored individual, interpersonal, organizational, community, and environmental multifaceted factors that influence smoking. Although there is a known correlation between poverty and smoking, there has not been a comparative analysis on a local level in this large urban area.

## Methods

**FIG. 1.**
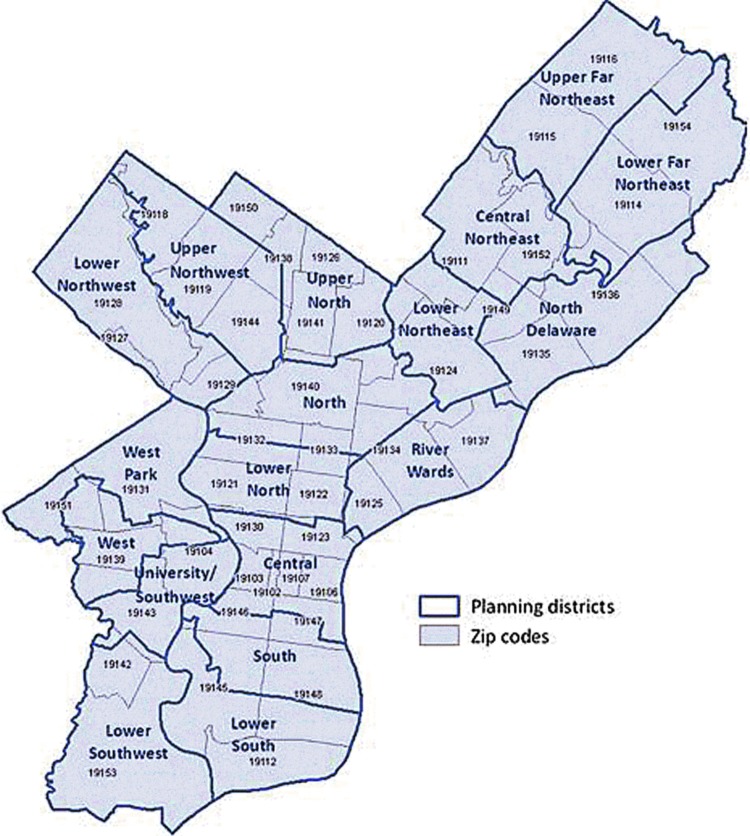
Philadelphia planning district map.

With preexisting borders and definitive social norms, planning districts within Philadelphia (created as part of Philadelphia 2035) are self-contained ecosystems that provide rich data that go beyond zip codes or census tracts^3,8^ (Figure 1). Studying planning districts in Philadelphia is necessary to create a city of health and equity. While examining these 18 districts, the research team noticed sharp differences in poverty rate, race, ethnicity, and public amenities. As such, the team felt these district boundaries would make for a realistic mechanism with established public data that would allow for the examination of impactful variables that relate to smoking in the city.

Poverty rates, adult smoking rates, district demographic statistics, and public amenity data were obtained from open sources, including the Philadelphia Department of Public Health, the 2012–2015 Public Health Management Corporation Household Health Survey, the Pennsylvania Department of Education, and the US Census Bureau.^1,2,11–13^ Data from the Public Health Management Corporation Household Health Survey were based on a representative sample of 5000 Philadelphians per biannual cycle between 2012–2015. US Census Bureau data were based on the American Community Survey, 5-year estimates that were published in 2015. The Philadelphia Department of Public Health culled data sets from the aforementioned sources and published Community Health Assessments in 2016 and 2017, which the research team utilized to conduct this analysis.

This paper will use adult smoking rate and smoking rate interchangeably and will define adult smoking rate as “the percentage of adults who have smoked at least 100 cigarettes in their lifetime and currently smoke every day or some days.”^1,2^ Pearson correlation was used to examine the association between planning district poverty rates and district smoking rates. Further, the researchers sought to identify multifaceted interactions and factors that influence Philadelphians' smoking behaviors utilizing the Social Ecological Model (SEM).

SEM is a framework used to examine multifaceted interactions between individual, interpersonal, organizational, community, and environmental factors and how they influence behaviors.^14^ This model takes into account multilevel factors related to all aspects of an individual's life and ensures that factors outside an individual that influence behavior are not ignored. The model allows for behaviors to be observed in the context of predetermined factors.^14^ Smoking is linked to poverty levels and race; however, one must not view the behavior of smoking simply as an individual's choice but as the result of several interrelated factors. SEM will be used to assess interactions between complex factors at all levels and how they influence smoking in Philadelphia's planning districts.

## Results

### Poverty and smoking disparities in Philadelphia planning districts

Philadelphia has the highest poverty rate of large cities and third worst income gap in the United States.^1,2,4^ This is clearly displayed by the 35.7% difference between the planning district with the lowest poverty rate and the district with the highest poverty rate.^1,2^ Planning district poverty rate is defined as the “percentage of the population, including all ages, living in a household with an income below 100% of the federal poverty level.”^1^

The highest planning district poverty rate is 45.4% in the North District, which is home to 137,849 individuals (Table 1).^2,15^ The majority minority district has a median annual income of $22,241 and only 2% of the population attended ≥4 years of college.^2,15^ The lowest planning district poverty rate is 9.7% in the majority white Lower Far Northeast District.^2,16^ This district has a population of 136,945, of whom 20% attended ≥4 years of college. The median annual income in the Lower Far Northeast is $55,478.^2,16^ To further highlight planning district disparities, only 2 have a poverty rate of less than 13%, while 5 districts have a poverty rate of more than 30%.^2^

**Table 1.** Descriptive Analytics of Planning Districts in Philadelphia

Planning district name	Population living 100% below the federal poverty level	Adult smoking rate	Smoking-attributable mortality rate per 100,000	Adults older than age 25 who completed some college	Adults who forwent health care because of cost in the past year	Adults who feel safe attending a local park during the day	Adults with a diagnosed mental health condition
Central	14.3%	16.1%	277.8	83.1%	10.7%	89.7%	16.3%
Central Northeast	16.3%	18.1%	352.5	43.2%	13.2%	71.7%	16.1%
Lower Far Northeast	9.7%	22.0%	422.9	47.8%	16.7%	77.3%	17.7%
Lower North	44.3%	25.3%	721.2	35.0%	11.4%	66.0%	28.9%
Lower Northeast	30.7%	23.3%	501.8	43.2%	17.2%	70.6%	15.0%
Lower Northwest	14.1%	16.9%	315.2	67.7%	10.0%	86.0%	16.5%
Lower South^a^	______	______	______	______	______	______	______
Lower Southwest	29.7%	26.4%^b^	472.2	41.7%	8.3%	69.9%^b^	13.9%
North	45.4%	28.3%	632.2	25.8%	19.8%	60.7%	26.4%
North Delaware	19.1%	26.4%	518.6	38.8%	16.6%	78.2%	19.6%
River Wards	29.3%	38.8%^b^	766.2	38.6%	25.7%^b^	74.7%	36.7%
South	22.2%	25.8%	597.3	43.9%	16.4%	74.3%	21.3%
University/Southwest	39.3%	21.8%	536.0	57.4%	19.5%	72.6%	22.5%
Upper Far Northeast	12.7%	9.4%	320.0	53.0%	10.2%	74.2%	13.4%
Upper North	24.3%	19.9%	578.8	44.0%	15.6%	62.7%	16.4%
Upper Northwest	24.0%	20.8%	455.5	60.5%	13.3%	81.9%	21.5%
West	34.9%	31.7%	510.9	42.9%	17.7%	70.0%	22.4%
West Park	28.1%	16.5%^b^	420.8	60.4%	Insufficient Sample Size	70.1%	Insufficient Sample Size

Data presented obtained from Philadelphia Department of Public Health, Community Health Assessment.^1,2^

^a^ Non-residential area

^b^ Small sample size

Similar to planning district poverty rates, adult smoking rates vary by district. The highest prevalence of smoking in the city is in the River Wards District at 38.8%, but it is worth noting the small sample size of this data point.^2^ This district is well known for containing Kensington, a neighborhood notorious for heroin. Following the River Wards District, the West District has the second highest smoking rate in the city.^2^ The third highest smoking rate in the city is in the North District. Notably, the North District has the highest poverty rate in the city.^2^ The lowest smoking rate is in the Upper Far Northeast District, a suburban area of the city containing neighborhoods such as Bustleton. This district has the second lowest poverty rate in the city.^2^ Conversely, the district with the lowest poverty rate in the city has the seventh highest smoking rate in Philadelphia^2^ (Table 1).

### Correlation between planning district poverty rates and adult smoking

The research team accessed publicly available data for planning district poverty rates, adult smoking rates, planning district resources, the racial breakdown of districts, and tobacco retailer density statistics in order to understand patterns and influential mechanisms that impact smoking in Philadelphia. Pearson correlation was used to examine the association between planning district poverty rates and district smoking rates among Philadelphians based on open access community health assessments.^1,2^ Results suggest a statistically significant association (*r* = 0.532; *P* = 0.028), indicating that as planning district poverty rates increase, district smoking rates also increase.

### Theoretical framework

**FIG. 2.**
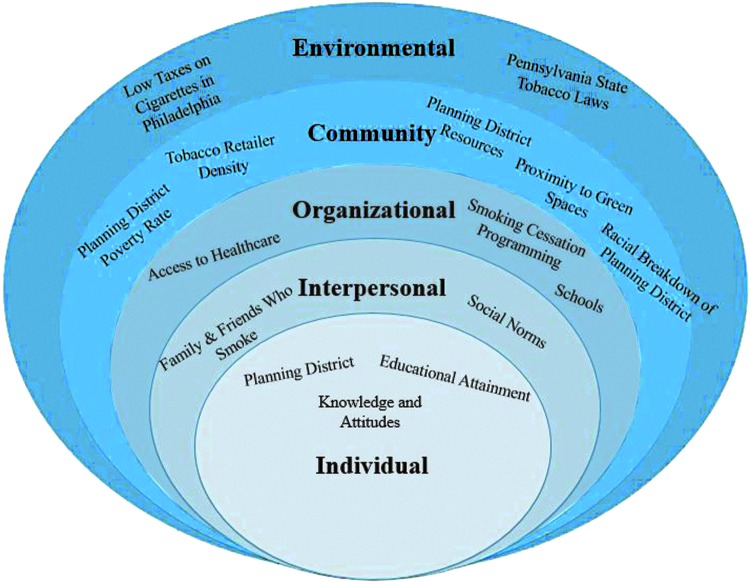
Social Ecological Model framework with key factors that influence smoking in Philadelphia.

Individual factors typically relate to knowledge, attitudes, behaviors, and personal traits.^14^ The planning district in which one lives is an individual-level factor; however, the planning district poverty rate is a community-level factor (Figure 2).

The planning district in which one lives can directly impact one's health and health behaviors through upstream and upper-level influences such as organizational, community, and environmental factors. Educational attainment is an individual-level factor that can influence smoking behaviors. Individuals without a high school diploma are more likely to smoke cigarettes than college graduates by a margin of 33%.^17^ Knowledge and attitudes toward smoking are key individual-level factors that can impact smoking. Both knowledge of the health risks and attitudes toward how serious risks are can influence smoking; those who put little emphasis on the risks of smoking are substantially more likely to smoke.^18^

Interpersonal factors include family, friends, and social networks.^14^ Interpersonal-level factors can be a key influence on smoking behaviors. Friends and family members who smoke is classified as an interpersonal factor. Literature suggests individuals with parents, friends, or extended relatives who smoke are more likely to begin smoking themselves.^19^ Social norms is another interpersonal factor that impacts smoking because smoking is a learned and socially mediated behavior.^20^

Organizational factors that can influence behavior are schools, institutions, and organizations.^14^ Organizational factors related to smoking are schools, smoking cessation programs, and access to health care. Living in a district that does not have hospitals or health care centers can influence smoking behaviors. Health care providers often are the first line of counseling patients on the health consequences of smoking as well as offering smoking cessation programming.^21^ Districts that do not have the ability to invest in school systems impact not only their residents' risks of smoking but also the educational attainment of individuals who live there, which also can influence smoking.^22,23^

Community factors include relationships between organizations, collaborative initiatives, and the community at large.^14^ Tobacco retailer density, planning district resources, proximity to green spaces, and racial breakdown of the district can all influence smoking behaviors at the community level. Tobacco retailer density in planning districts can influence smoking; in fact, high levels of tobacco retailer density can influence smoking behaviors and the number of cigarettes smoked each day.^22^

Planning district resources affect smoking; neighborhoods with a lower number of resources have higher rates of smoking than those with a high number of resources.^17,24^ Proximity to green spaces is a community-level factor that impacts smoking behaviors because access to green spaces helps alleviate stress, thus reducing the risk of smoking.^25,26^ Finally, the racial breakdown of the planning district has a bearing on smoking behaviors as some racial minorities have higher rates of smoking than whites.^27^

National, state, and local laws as well as policies are key environmental factors.^14^ Philadelphia must follow Pennsylvania state-level tobacco laws. The state mandates an individual must be at least 18 years of age in order to purchase tobacco products. In 2016, Pennsylvania mandated a tax of $2.60 per pack of cigarettes. The tax rate is high compared to the south; for example, in Georgia the tax is $0.37 per pack. However, Pennsylvania's tax is the lowest of the northeastern states; in New York the tax is $4.35 per pack^28,29^ (Table 2). This low rate of tax can influence smoking as it increases access to cigarettes. Philadelphia attempted to create an additional tax on cigarettes in an effort to address the high citywide smoking rate; however, the state legislature took away the city's ability to regulate tobacco sales and laws.^30^

**Table 2.** Cigarette Tax Rates by State

State	Geographic region	State rank (1 is the highest and 50 is the lowest)	Tax per 1 pack of cigarettes
New York	Northeast	1	$4.35
Rhode Island	Northeast	3	$4.25
Washington	Pacific Northwest	9	$3.03
California	West Coast	8	$2.87
Pennsylvania	Mid-Atlantic	11	$2.60
Illinois	Mid-West	19	$1.98
Texas	South-Central	27	$1.41
Mississippi	South	38	$0.68
Georgia	South	48	$0.37
Missouri	Mid-West	50	$0.17

Tax rates were as of 2018.^29^

### Linkages between planning district poverty rates and smoking rates

**FIG. 3.**
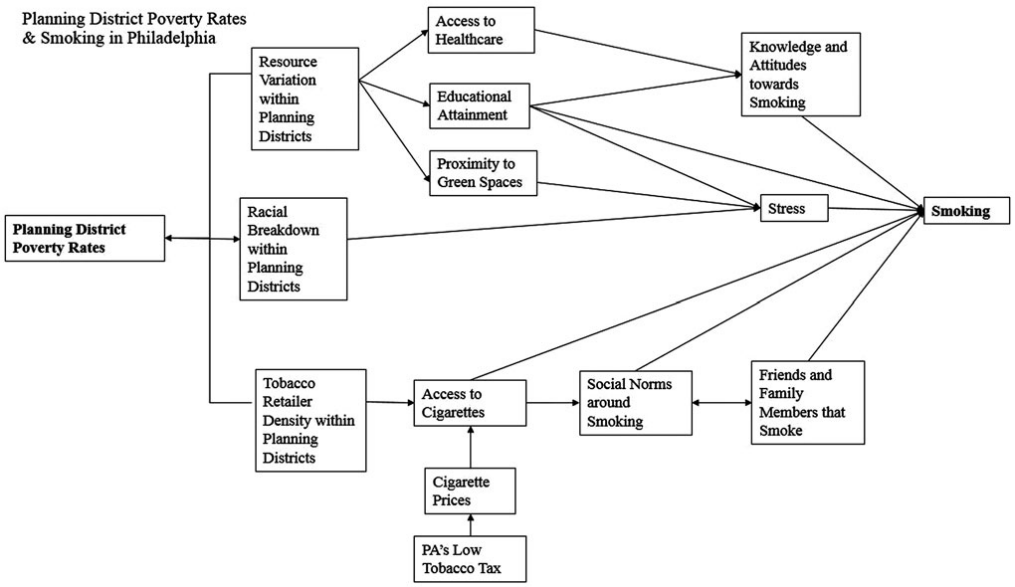
Casual diagram illustrating key impacts, factors, and proximal causes of smoking in Philadelphia.

The relationship between planning district poverty rates and smoking rates may contain hidden mediating factors, which this paper will seek to explore. There are several possible mechanisms linking planning district poverty rates and adult smoking rates in Philadelphia. A causal diagram was created to display potential links between planning district poverty rates and smoking (Figure 3). In the literature there is a clear association between poverty rates and smoking rates.^24^

### Impact one: resource variation within planning districts

The first impact of this pathway is planning district resources – a community-level factor. Factors under this impact include access to health care, educational attainment, and proximity to green spaces. Planning districts with high poverty rates have fewer resources than districts with lower rates of poverty.^31^ Living in a planning district with few resources can be linked to limited financial resources and a lack of opportunities. Poverty-laden districts have higher rates of unemployment, a lack of residential mobility, less cohesion, and higher rates of crime.^31^

Planning districts with fewer resources are more likely to have residents who are unable to access health care, an organizational factor. Health care providers play a pivotal role in educating patients about the risks of cigarettes and shaping their attitudes toward smoking. Physicians are often at the front lines of promoting smoking cessation and providing resources for patients, although literature debates whether the medical community provides enough cessation information.^21^

Providing residents with readily accessible health care is imperative for them to learn about the risks of smoking as well as the benefits of quitting. Access to health care is linked to knowledge and attitudes toward smoking, a proximal cause of smoking. As physicians play a key role in educating their patients on the risks of smoking as well as promoting the benefits of smoking cessation, it is clear that accessing health care directly influences patient attitudes and behaviors. Individuals who put less emphasis on the health risks of smoking are more likely to begin smoking.^18^ This dearth of emphasis on the risks of smoking can be tied to a lack of knowledge of the risks.

In Philadelphia's North District, the poorest district, with the third highest rate of smoking, 16.6% of adults reported they did not have health insurance.^2^ This is the second highest rate of uninsured adults in the city. Furthermore, this district has the second highest number of adults who forgo health care because of cost, 19.8%, and the highest percentage of adults covered by Medicaid, 40.9%.^2^

A lack of resources in a planning district can be linked to schools with the inability to invest in their students. Schools in districts with high poverty rates do not have the resources they need to provide students with an adequate education and tend to produce persons with lower levels of educational attainment, an individual-level factor.^23^ Students living in poverty-stricken districts reach fewer education milestones.^1,2,23^ The North District has the lowest level of educational attainment, with 27.8% of adults older than age 25 completing some college, in addition to being the poorest district with the third highest rate of smoking.^2^

Educational attainment is linked to 2 proximal causes of smoking, knowledge and attitudes as well as stress, both individual-level factors. Data suggest that educational attainment can be considered a proximal cause itself; those with lower levels of educational attainment have a higher risk of smoking.^17^ Adults who completed less than a high school diploma are 33% more likely than college graduates to smoke, and those with less than a high school diploma have less success with smoking cessation than those with higher levels of education.^17^

Philadelphia has poor rates of educational attainment. Nationally, 82.3% of students graduate from high school within 4 years, yet only 65% of Philadelphians do.^2^ This low level of educational attainment may influence the high smoking rates in the city as smoking is correlated with level of education.^17^

Educational attainment can influence the proximal cause of smoking – knowledge and attitudes. Education impacts the knowledge and attitudes one has regarding smoking. Individuals with higher education levels have better knowledge of the health risks associated with smoking.^32^ Further, smoking behaviors and attitudes are correlated with educational attainment.^17,33^ Persons with lower levels of education recalled health warnings on cigarette packages far less frequently than those with higher levels.^33^ People who live in planning districts where they are less likely to achieve at least a high school diploma are at greater risk for smoking.^17^ Those with higher education levels are more likely to have negative attitudes toward smoking and view it as a risky behavior.^18,32^

Educational attainment also can impact stress, which is a proximal cause of smoking. People often begin smoking and continue the behavior because of stress.^34^ Smoking is cited as a way to relieve stress and those who live in a resource-poor district are more likely to use cigarettes as a means to unwind.^34^ Sources of stress related to educational attainment include discrimination, lack of access to health care, poor housing conditions, trouble making ends meet, and work-related problems.^35^ Individuals with low levels of educational attainment experience higher levels of work stress as well as chronic stress and are more likely to smoke as a coping mechanism.^34,35^ Further, they are more likely to live in chaotic districts, with high population density, noise, and a lack of access to health care – all of which can increase stress levels.^35^

The third factor related to planning district resources is the community-level factor, proximity to green spaces. Planning districts that do not offer easy access to a green space leave residents at risk for an inactive lifestyle.^36^ A lack of access to green space can cause an increase in stress as individuals are unable to get outside, engage in physical activity, or decompress.^25^ Therefore, proximity to green spaces is linked to stress, a proximal cause of smoking.

Many smokers utilize cigarettes as a means to relax and unwind.^26,34^ Without the ability to decompress from stress through accessing green spaces and outdoor parks, residents in planning districts may turn to cigarettes to relieve stress. The North District, the poorest district in the city with one of the highest rates of smoking, has the highest rate of residents without access to a green space. Only 60.7% of residents in the planning district feel safe walking to a public park or outdoor space during daylight hours, the lowest rate in the city.^2^

### Impact two: racial breakdown within planning districts

The second impact of the pathway is the racial breakdown of a planning district, which is a community-level factor. The racial breakdown of districts and the poverty rate have a reciprocal relationship because they influence one another. Racial minorities in Philadelphia are more likely to live in poverty than whites. Nearly 40% of Hispanic Philadelphians live in poverty, followed by 31.5% of blacks, 26.3% of Asians, and 17.8% of whites.^2^ Not only are minorities more likely to live in poverty, but planning districts with high poverty rates have more minority residents. The poorest planning district in Philadelphia is 46% black and 21% white compared to the planning district with the lowest poverty rate, which is 58% white and 18% black.^2,15,16^

The racial breakdown of planning districts is a relevant factor in smoking rates because smoking tends to be higher in minority populations.^27^ Moreover, blacks struggle with smoking cessation more than whites, despite attempting to quit more frequently.^37^

The racial breakdown of planning districts is linked directly to stress. Minority individuals experience discrimination and an increased level of stress compared to nonminority individuals.^38^ The fact that minorities experience higher levels of discrimination and stress is compounded by the fact that many minorities in Philadelphia live in poverty.^2^ Low income and socioeconomic status can contribute to stress, depression, and overall poor psychological health, which can trigger a breakdown in physical health.^31^

The majority minority North District is not only the poorest planning district, but also has the third highest rate of adults with a diagnosed psychological condition.^2^ Persons living in poverty have more stressors than the general population and existing data state that many individuals use smoking as a coping mechanism to escape from daily stressors and seek pleasure.^26,34^ Therefore, it can be postulated that minority individuals, who already experience a disparate amount of stress and discrimination, which is then exacerbated by living in poverty, may engage in smoking in order to relieve their stress.^26,38^

### Impact three: tobacco retailer density within planning districts

The third impact of this pathway is tobacco retailer density, which is a community-level factor. Tobacco retailer density is comprised of factors that include access to cigarettes, cigarette prices, Pennsylvania's low tobacco taxes, social norms around smoking, and family members and friends who smoke. Tobacco retailer density varies based on planning district poverty rates. Planning districts with higher rates of poverty have disproportionately more tobacco retailers. In fact, the per capita tobacco retailer density in planning districts with high poverty rates is 69% higher than in districts with low rates of poverty.^39,40^ “Big tobacco” intentionally puts more retailers and advertisements in neighborhoods with high poverty rates.^17^

Planning districts with high rates of poverty are targeted for special discounts and deals geared toward individuals of low socioeconomic status.^17^ The poorest district in Philadelphia has the highest tobacco retailer density in the city with an average of more than 3 retailers per 1000 residents, whereas the Lower Far Northeast – the district with the lowest poverty rate – has 0.67 tobacco retailers per 1000 residents.^39,40^

Access to cigarettes can be influenced by tobacco retailer density because more stores cause more competition and lead to a drop in cigarette prices.^17^ Retailers may be incentivized to run special deals, which occur disproportionately in high poverty districts.^17^ Furthermore, if there are many retailers selling tobacco products, it is easier and more convenient to purchase cigarettes.^17^ Living in close proximity to tobacco retailers can influence smoking behaviors and the number of cigarettes smoked each day.^22^ Access to cigarettes also can be considered a proximal cause of smoking because easier accessibility increases the likelihood of smoking.^10^ Philadelphia has noted that the ease of accessibility to tobacco products increases the number of smokers in the city.^10^

Cigarette prices are inexpensive in Pennsylvania compared to surrounding states.^29^ This is because of a statewide tax policy, which is an environmental factor.^28^ Philadelphia has linked the cheap prices of cigarettes to the high rates of smoking in the city, which motivated the city to attempt to implement a citywide additional tax policy. However, as previously stated, state government stripped the city of the power to regulate tobacco taxes.^10,30^

Social norms, an interpersonal-level factor, can be influenced by the number of tobacco retailers. Readily available cigarettes in Philadelphia can impact social norms and normalize smoking behaviors. Smoking is a socially normative behavior, making social norms a key influence on one's likelihood of starting to smoke.^20^ Therefore, social norms around smoking can also be considered a proximal cause of smoking. Smoking is a learned behavior and is socially mediated.^20^ People experiment with smoking to cultivate a social identity. Tobacco is seen as a luxury and people who smoke are viewed as cool and more attractive.^20^ These social norms impact the likelihood of individuals smoking.

Friends and family members who smoke is an interpersonal factor linked to tobacco retailer density. Planning districts with a high number of tobacco retailers are likely to have residents living in them who know a smoker. Having friends and family members who smoke is a factor influenced by social norms. Norms around smoking that are more lax increase the likelihood of knowing someone who smokes. This relationship works the other way as well; knowing someone who smokes will impact one's perception of the social norms around smoking. Individuals who have smokers in their social circle have an increased risk of smoking themselves.^19^ Based on this, having friends and family members who smoke should be considered a proximal cause of smoking.

## Discussion

This study found that planning district poverty rates in Philadelphia parallel smoking rates among residents. Within the city borders, planning district poverty rates are correlated with smoking rates (*r* = 0.532; *P* = 0.028). Based on this investigation, the research team hypothesizes that elements that influence smoking rates in Philadelphia are not limited to poverty rates alone, but include multilevel factors such as educational attainment, stress, planning district resources, and insufficient tobacco control measures.

Planning districts that have a higher level of poverty rates are the same districts that have high rates of adult smoking. For example, the North District has a high poverty rate and smoking rate whereas the Upper Far Northeast has low rates of poverty and smoking.^1,2^ Planning districts with high poverty rates and high smoking rates also have lower levels of educational attainment, less access to green spaces, and higher rates of diagnosed psychological conditions.^1,2^ These patterns imply that several multifaceted factors influence an individual's smoking behavior. Planning district poverty rates appear to directly and indirectly influence the rate of smoking of the residents in each district.

The SEM framework is well suited to investigate how Philadelphia's planning district poverty rates, a community-level factor, influence smoking rates of residents, as smoking is a behavior that is influenced by numerous multidimensional factors. Although many of the factors related to SEM constructs are not proximal causes of smoking, they still carry weight and influence smoking behavior. Because smoking is a complex behavior, it is important to examine factors beyond poverty and SEM provides a strong framework for this analysis. Philadelphia must address not only the extremely high rate of smoking but also the rapidly increasing number of disparities in health, income, education, and access to health care in each district.

### Limitations

This analysis utilized group-level/planning district data, thus making it difficult to make inferences about the individuals living in each planning district. Although aggregated data proves useful for ecological studies, it can lead to the loss or concealment of important data points that may exist in an individual-level analysis. Therefore, further investigation into the relationship between poverty and smoking in Philadelphia is warranted to avoid a potential ecological fallacy. Despite this fallacy, the researchers remain confident in their suggested strategies for the city to take up.

### Future directions

Tackling the high rates of smoking in Philadelphia will not be a small task. In order to mitigate the disparate rates of smoking based on planning district poverty rates, the city must address a long list of systematic inequalities. Philadelphia 2035 – a plan to boost the economy, promote a healthy population, and embrace environmentally friendly practices in the city – has goals that potentially can address these disparities, including boosting the economy and promoting a healthy population in each planning district.^3,8^

To reduce smoking, Philadelphia must invest resources in each planning district, allotting more funding to districts with high poverty rates and few resources. Increasing the resources in planning districts with high poverty rates will allow residents to access health care, promote physical activity and mental well-being through greenspaces, as well as provide rigorous education for students, allowing them to be properly set up for a bright future.

Attempts to create new social norms around being healthy and the importance of smoking cessation or never picking up a cigarette can contribute to the goal of reducing smoking in the city. Although challenging long-standing social norms will be difficult, Philadelphians will benefit in the long run.

Finally, Philadelphia should take legal action and petition the state to allow the city to raise tobacco taxes, or lobby for the entire state to raise taxes on cigarettes. The city also should create new laws that forbid new tobacco retailers and limit the number per planning district, regardless of poverty rate.

Although the focus of this analysis was to explore the linkage between poverty and smoking in Philadelphia as well as to examine smoking through the lens of SEM, it is worth noting that poverty is a multifaceted problem and also can be contextualized using SEM. Several of the strategies suggested to decrease rates of smoking will have an impact on poverty rates as well. Investing a greater number of resources in poverty-stricken planning districts will provide an opportunity for education and increased access to health care.

Low levels of educational attainment are correlated with smoking behaviors as well as an inability to make ends meet and an increase in stress levels.^32,35^ Creating sustainable education opportunities for low-income residents in Philadelphia would not only reduce smoking rates, but would reduce poverty rates by creating an increase in employment prospects.

Additional resources should be provided to fund low-cost clinics in order to increase access to health care. Access to health care can influence one's risk of smoking.^18^ As demonstrated by the health–wealth gradient, poverty corresponds to health status and increases the risk of engaging in harmful health behaviors.^6^ By improving health care access, it would allow Philadelphians to improve their health and thus their economic security. Furthermore, reducing poverty rates would decrease smoking rates as well as create a potential ripple effect for other health behaviors.

Philadelphia must address both smoking and poverty rates through investment in its planning districts, which may prove costly. A citywide tax increase on tobacco products could create additional revenue for the city to use in order to advance the well-being of its residents by improving education and health care, as well as address long-standing systematic inequalities throughout planning districts.

Philadelphia is rich in history and culture, but has marked disparities in health behaviors. The city is making strides toward equity for all residents. Smoking rates and poverty rates should be tracked over the next decades to measure progress. In creating an economically and physically healthy city, it will be necessary to address: (1) planning district poverty rates; (2) planning district resources including access to health care and green spaces, and improving education; (3) residents' stress, especially minorities or those who live in a district with a dearth of public amenities; and (4) tobacco retailer density through implementing higher taxes on cigarettes, limiting the number of retailers, and reducing access to tobacco products, which may create new social norms around smoking while reducing the number of smokers an individual knows.

With time, such an approach can further reduce the smoking rates, which recently have plateaued throughout the city. If we are to realize a future in which smoking and smoking-related diseases are dramatically reduced, a comprehensive approach considering the SEM framework will be required. The research team believes that such an approach is mandated to improve the health of residents of the City of Philadelphia.
